# Cost-effectiveness of talazoparib for patients with germline BRCA1/2 mutated HER2-negative advanced breast cancer in China and the US

**DOI:** 10.1038/s41598-024-64343-7

**Published:** 2024-06-17

**Authors:** Junjie Pan, Ning Ren, Lanqi Ren, YiBei Yang, Qiaoping Xu

**Affiliations:** 1 Fourth Clinical Medical College of Zhejiang Chinese Medical University, Affiliated Hangzhou First People’s Hospital, Hangzhou, 310053 China; 2 Department of Clinical Pharmacology, Key Laboratory of Clinical Cancer Pharmacology and Toxicology Research of Zhejiang Province, Cancer Center, Affiliated Hangzhou First People’s Hospital, Westlake University School of Medicine, Hangzhou, 310006 China

**Keywords:** Talazoparib, Cost-effectiveness, Germline BRCA-mutated, Markov model, Breast cancer, Cancer, Diseases

## Abstract

Breast cancer is one of the tumors with the highest prevalence rate among women in the world, and its BRCA1/2 gene is a common mutation site. Talazoparib, as a targeted PARP inhibitor, can effectively control the occurrence and development of breast cancer with BRCA1/2 gene mutation, and play a therapeutic role. Based on the findings from the Phase III EMBRACE trial (NCT01945775 clinical trial), our analysis reveals that the talazoparib group demonstrated a significant extension in progression-free survival, along with improved response markers and patient-reported outcomes when compared to conventional therapies. This study aims to assess the cost-effectiveness of talazoparib for treating advanced breast cancer with germline BRCA1/2 mutations and HER2 negativity, considering the perspectives of health services in China and the United States. The results obtained will serve as a valuable reference for promoting rational drug utilization and enhancing medical resource efficiency. To evaluate the cost-effectiveness of Talazoparib more scientifically and provide clinicians with chemotherapy options, this paper developed a Markov model based on the EMBRACA clinical trial (clinical Trails.gov No., NCT01945775) to simulate the survival events of breast cancer patients in the Talazoparib group and the standard treatment group. The state transition probability and clinical data of breast cancer patients during treatment were extracted from the phase III EMBRACA clinical trial. The cost data generated during the treatment process comes from local hospital pricing, other references, and expert consultation. This article uses US dollars to calculate the treatment cost and incremental cost-effectiveness ratio. Health outcomes are expressed in Quality Adjusted Life Years (QALYs). In addition, Outcomes were measured in quality-adjusted life-years (QALYs), and incremental cost-effectiveness ratio, which robustness was evaluated by deterministic and probabilistic sensitivity analyses. This article establishes a Markov model for single-item sensitivity analysis. The results show that the economic benefits of using Talazoparib as a new treatment strategy in both China and the United States are higher than other drugs, and it is cost-effective. Compared to the control group, the incremental cost incurred by the Talazoparib treatment group in China was $2484.48/QALY, with an incremental QALY of 1.5. However, Talazoparib in the United States holds a dominant position, saving costs of $10,223.43 and increasing QALYs by 1.5. The clinical treatment effect of Talazoparib group in BRCA1/2 mutant advanced breast cancer patients is better than that of the standard treatment group, and the progression free survival period is significantly prolonged. From the perspective of medical and health services in China and the United States, the Talazoparib group is more economical than the standard treatment group in treating patients with BRCA1/2 mutant advanced breast cancer.

## Introduction

Breast cancer is one of the diseases that women often suffer from, and it is also a common cause of cancer death in women^1^. Breast cancer has become the fifth leading cause of cancer-related deaths. About 25% of female cancer patients worldwide have suffered from breast cancer, and the number of deaths due to breast cancer has reached about 11% of the total number of female cancer deaths worldwide^2^. Among the genes related to the occurrence and development of breast cancer, the BRCA1/2 gene is the earliest and most studied gene. The study found that 10% of breast cancer patients' cancer cells had BRCA1/2 gene mutations, and patients with BRCA1/2 gene mutations were more likely to suffer from breast cancer than normal people^3,4^. The BRCA breast cancer susceptibility gene, as a tumor suppressor gene, repairs broken double-stranded DNA mainly through homologous recombination and repair, to prevent the unstoppable proliferation of cells caused by canceration. BRCA1 and BRCA2 are located on chromosomes 17q21 and 13q2, respectively. Their division of labor in cells is not the same, with BRCA1 having the function of repairing DNA damage and activating checkpoints, while BRCA2 can promote homologous recombination of chromosomes^5^. The abnormal mutation of breast cancer susceptibility gene 1 or 2 (BRCA1/2) in breast cancer cells cannot repair DNA double-strand breaks but relies on the DNA single-strand repair pathway regulated by adenosine diphosphate ribose polymerase (PARP). By inhibiting PARP, the damaged DNA cannot be effectively repaired, thus inducing the death of breast cancer cells with BRCA1/2 gene mutation. In addition, PARP inhibitors can capture PARP in damaged DNA and accelerate cell death^6,7^. Poly (ADP ribose) polymerase inhibitor can effectively play an anti-tumor role in BRCA mutant ovarian cancer and breast cancer. As the first one to treat PARPi-negative breast cancer, olaparib caused drug resistance in breast cancer due to its high expression of P-glycoprotein and breast cancer drug-resistant protein. Talazoparib has become the most effective PARPi for the treatment of locally advanced BRCA mutation or metastatic HER2-negative breast cancer^8^. In Phase III clinical trial^6^, In the treatment of advanced breast cancer patients with BRCA mutation in the reproductive system, the use of Talazoparib shows the anti-tumor activity of Talazoparib. In the reported outcomes of patients, the overall health status and quality of life of the Talazoparib group were significantly improved compared with the standard treatment group and effectively delayed the malignant progression of breast cancer. The disease or death risk ratio for the Talazoparib group and the standard treatment group was 0.54, the median risk ratio for mid-term mortality was 0.76, the objective response rates were 62.6% and 27.2%, respectively, and the median progression-free survival was 8.6 months and 5.6 months, respectively^6^. According to the results of this trial, Talazoparib is more effective than standard treatment for patients with BRCA1/2 mutant advanced breast cancer. There is no evidence on whether Talazoparib is cost-effective in the treatment of BRCA1/2 mutant breast cancer, and there is no scientific conclusion on its cost. Cost–benefit analysis is the process of comparing the cost and effectiveness of various regimens to determine whether their therapeutic value is superior. This study established a Markov model to analyze the cost-effectiveness score of Talazoparib in treating BRCA1/2 mutant breast cancer in China and the US. It provides an economic basis for clinical medication and treatment decisions for scientific breast cancer treatment.

This study evaluates the cost-effectiveness of talazoparib vs. the physician’s choice of chemotherapy as maintenance therapy for germline BRCA1/2 mutated HER2-negative advanced breast cancer from the perspective of healthcare in China and the US. The results provide a reference for patients with BRCA mutation and metastatic breast cancer in both countries to choose a safer, more effective and more economical clinical treatment scheme.

## Method

### Model overview

**Figure 1:**
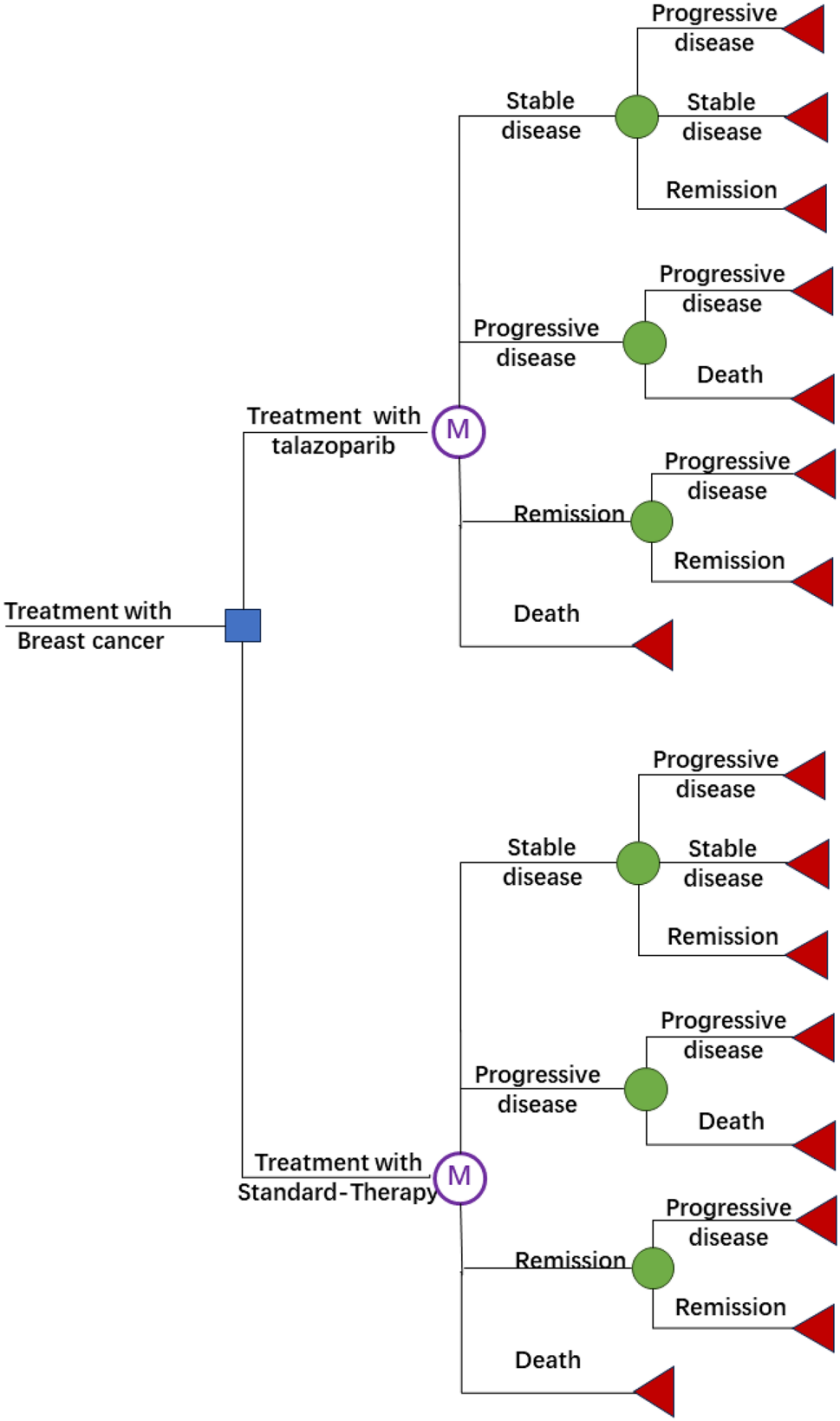
Transition probability calculation procedure.

We use TreeAge Pro Suite 2022 (TreeAge Software Company, Williamstown, MA, USA) to construct decision trees and Markov models^9^. We designed a patient population based on the NCT01945775 experiment, including eligible patients in our model, and randomly assigned them in a 2:1 number to receive the Talazoparib regimen or standard treatment regimen for treatment. Then, cost-effectiveness analysis was conducted using overall survival OS, progression-free survival PFS, duration of remission DOR, and relative risk RR as indicators. Before receiving treatment, the patient is in a different health state. After receiving treatment, the patient changes four health states (stable (SD), progressive (PD), remission (RE), and death (DE)) until entering the final state of death, with a period of 5 years. (Fig. 1) The entire cycle length is set to 21 days, which is equivalent to the duration of a chemotherapy cycle. The result parameters we measured include total cost, QALY, incremental cost-effectiveness ratio (ICER), and net monetary benefit (NMB). Among them, cost and survival rate are estimated using semi-circular correlation and calculating an annual discount rate of 5%^10^. The calculation method for incremental cost-effectiveness is to divide the difference between two treatment plans and the corresponding utility value of the two treatment plans^11^.

According to the standards of the Chinese Pharmacoeconomics Evaluation Guidelines, this study uses the per capita gross domestic product (GDP) and triple the per capita GDP of China and the US as thresholds and evaluates the cost-effectiveness of the Talazoparib treatment strategy by comparing the numerical values between ICER and per capita GDP. If ICER is lower than the per capita GDP, then this strategy is cost-effective; If the ICER is between three times the per capita GDP and per capita GDP, then this strategy is also cost-effective and acceptable; If the ICER is greater than three times the per capita GDP, then this strategy has no economic value. Willingness to pay (WTP) level is the maximum treatment cost that a patient is willing to pay to obtain an additional quality-adjusted life month. In 2022, China's per capita GDP reached $12,741, while the per capita GDP of the US reached $76,400. Due to the one-month cycle in the Markov model of this study, we set the WTP thresholds for China and the US at $3185/month and $19,100/month, respectively.

A total of 431 patients with advanced breast cancer were included in the NCT01945775 trial. The inclusion criteria used in this trial are as follows: (1) Age ≥ 18 years old, not suitable for radical local advanced or metastatic breast cancer (2) Existence or suspicion of BRCA1/2 mutation. (3) Accepting less than or equal to three types of cytotoxic drugs, having previously received treatment with paclitaxel, anthracyclines, or both, there are no contraindications. (4) It can be included in previous platinum based adjuvant therapy, provided that it is in the disease-free interval for 6 months after the last administration. (5) When platinum chemotherapy was excluded, advanced breast cancer progressed. Patients with advanced breast cancer were randomly divided into two groups at a ratio of 2:1. Among them, a total of 287 people in the talazoparib group received treatment with talazoparib (1 mg, once daily), while 144 people in the standard treatment group were assigned to receive standard treatment. The standard treatment consisted of capecitabine (1.25 g/m^2^, twice daily for two weeks), eribulin (1.4 mg/m^2^, administered once on days 1 and 8), gemcitabine (1 g/m^2^, once a week), and vinorelbine (25 mg/m^2^, administered once on days 1 and 8), accounting for 44%, 40%, 10%, and 7% of the standard treatment group patients, respectively (rounded to a total percentage of > 100%). The baseline data of patients is shown in Table 1.

**Table 1:** Baseline cohort characteristics.

	Talazoparib (n = 287)	Standard-Therapy (n = 144)
Mean (SD) age, years	47.5	49.4
Female. N(%)	98.6	97.9
ECOG performance status score—%^†^		
0	53.3	58.3
1	44.3	39.6
2	2.1	1.4
< 12-mo disease-free interval from initial diagnosis to advanced breast cancer—no. (%)	108(37.6)	42(29.2)
Breast cancer stage—no. (%)^‡^		
Locally advanced	15(5.2)	9(6.2)
Metastatic	271(94.4)	135(93.8)
BRCA status—no. (%)^§^		
BRCA1-positive	133(46.3)	63(43.8)
BRCA2-positive	154(53.7)	81(56.2)
Hormone-receptor status—no. (%)		
Triple-negative	130(45.3)	60(41.7)
Hormone-receptor–positive	157(54.7)	84(58.3)

^†^The Eastern Oncology Collaborative Group (ECOG) Physical Status Scale in the US has a score range of 0–5, where 0 indicates the patient has no symptoms, while a higher score indicates more severe symptoms.

^‡^One patient in the talazoparib group experienced data loss.

^§^Six patients in the talazoparib group and four patients in the standard treatment group were diagnosed with suspected harmful mutations in the BRCA1/2 gene, while all other patients were diagnosed with harmful mutations in the BRCA1/2 gene.

### Model probabilities

The transition probability of Markov state models refers to the probability of a patient transitioning from one of the four states to another during a treatment cycle in pharmacoeconomic evaluation. In this study, we use the Deale method to analyze the incidence rate and various survival data and use a simple exponential function to approximate the expected life^12,13^. Among them, the four clinical efficacy indicators of RR, OS, PFS, and DOR are an important basis for calculating the probability of metastasis. The data required for transfer probability is sourced from the NCT01945775 experiment, and its calculation method is shown in Table 2.

**Table 2:** Key parameters of Talazoparib and Standard Therapy in the treatment of the metastatic breast cancer model.

Variable/Probability	Formula	Best estimate	SA range	Distribution	Source
Talazoparib					^6^
RR		0.646			
OS		24.3			
PFS		8.6			
DOR		5.4			
Stable → Stable (Tss)	1-Tsp-Tsr	0.443	0.3544–0.5316	Beta	
Stable → Remission(Tsr)	1-exp (− RR/3)	0.193	0.1549–0.2325	Beta	
Stable → Relapse(Tsp)	Trp*4	0.364	0.2937–0.4406	Beta	
Remission → Remission(Trr)	1-Trp	0.909	0.7266–1.0000	Beta	
Remission → Relapse(Trp)	1 − exp(− 0.75*In(2)/(DOR))	0.091	0.0734–0.1101	Beta	
Relapse → Relapse(Tpp)	1-Tpd	0.968	0.7739–1.0000	Beta	
Relapse → Death(Tpd)	1 − exp(− 0.75*In(2)/(OS-PFS))	0.032	0.0260–0.0391	Beta	
–Standard-Therapy					^6^
RR		0.111			
OS		6.3			
PFS		5.6			
DOR		3.1			
Stable → Stable (STss)	1-STsp-STsr	0.347	0.2776–0.4164	Beta	
Stable → Remission (STsr)	1-exp(− RR/3)	0.036	0.0290–0.0436	Beta	
Stable → Relapse(STsp)	STrp*4	0.617	0.4940–0.7411	Beta	
Remission → Remission (STrr)	1-STrp	0.846	0.6765–1.0000	Beta	
Remission → Relapse (STrp)	1 − exp(− 0.75*In(2)/DOR))	0.154	0.1235–0.1853	Beta	
Relapse → Relapse (STpp)	1-STpd	0.476	0.3807–0.5710	Beta	
Relapse → Death(STpd)	1 − exp(− 0.75*In(2)/(OS-PFS))	0.524	0.4193–0.6289	Beta	
Discount rate for costs and QALYs		3% per year			
Health state utilities					
No recurrence (chemotherapeutic period)		0.74	0.0592–0.888	Beta	
No recurrence(after chemotherapy)		0.94	0.752–1.000	Beta	
local recurrence (in the first year)		0.74	0.592–0.888	Beta	
remission		0.85	0.680–1.000	Beta	
Relapse		0.5	0.400–0.600	Beta	

RR = (OS − PFS)/OS.

OS refers to the time of patient death from the randomized grouping value.

PFS refers to the time from when the subject enters the experiment to when the tumor progresses or dies.

DOR is the time from the first assessment of CR or PR to the first assessment of progression or death.

This paper conducts a corresponding cost–benefit analysis for Chinese and Americans to determine the economic benefits of Talazoparib for patients with advanced breast cancer in both countries. During the Talazoparib and Standard Therapy subgroup analysis, median FPS (8.6 months and 5.6 months), median OS (24.3 months and 6.3 months), and DOR (5.4 months and 3.1 months) were obtained.

### Cost and health state utility values

The cost calculated in this study encompasses the expenses associated with chemotherapy drugs, adverse reaction treatment, routine care, laboratory tests, routine follow-up, CT scans, and BRCA typing tests. However, it does not include bed fees, injection fees, or other miscellaneous charges. The cost of treatment considered in the model was computed based on drug treatment cycles. All fees generated by the model were converted into US dollars using the 2022 Sino-US currency exchange rate. Calculate the total cost of treatment and obtain the ICER. If ICER is greater than WTP, the Talazoparib group is not cost-effective in treating advanced breast cancer patients with BRCA1/2 mutations. If the ICER is less than WTP, the Talazoparib group is cost-effective in treating advanced breast cancer patients with BRCA1/2 mutations. Based on clinical data, we included 5 common adverse reactions. To calculate the drug dosage, it is assumed that the average surface area of Chinese patients is 1.67 m^2^, while the average surface area of American patients is 1.72 m^2^. The prices of various medical expenses are sourced from relevant public literature, local hospitals, and pharmaceutical intelligence websites. The relevant cost data is shown in Tables 3 and 4.

**Table 3:** Cost in China.

Parameter name	Base ($)	Range ($)	Distribution	Source
Medication costs				
PFS				
Talazoparib(1 mg)	163.85	131.08–196.62	–	www.yaozh.com
Talazoparib(cycle)	3440.97	2752.78–4129.16	Triangular	www.yaozh.com
Eribulin(1 mg)	547.12	545.49–654.59	Triangular	www.yaozh.com
Eribulin(cycle)	2481.76	2413.04–2,895.64	–	www.yaozh.com
Gemcitabine(200 mg)	5.87	4.70–7.05	Triangular	www.yaozh.com
Gemcitabine(cycle)	1017	139.23–167.07	–	www.yaozh.com
Capecitabine(500 mg)	4.65	3.72–5.58	Triangular	www.yaozh.com
Capecitabine(cycle)	9.31	7.45–11.17	–	www.yaozh.com
Vinorelbine(10 mg)	18.89	15.11–22.67	–	Tertiary hospitals
Vinorelbine(cycle)	170.03	136.02–204.04	–	Tertiary hospitals
BRCA 1/2 mutation profiling	1,378.58	1102.87–1654.30	Triangular	yycg.hnsggzy.com
PD				
Docetaxel (0.5 mL: 20 mg)	110.62	99.55–121.68	–	www.yaozh.com
Docetaxel (per cycle)	884.92	796.42–973.41	–	www.yaozh.com
Prednisone (per cycle)	0.52	0.30–0.572	–	www.yaozh.com
Non-pharmaceutical cost				
Laboratory testing (cycle)	75.47	67.92–83.02	Gramma	^14^
CT (per cycle)	47.23	42.51–51.95	–	^14^
Nursing fee (per cycle)	184.82	166.33–203.30	–	^14^
Routine follow-up (per cycle)	7.05	6.34–7.75	Gramma	^14^
Cost of treatment of adverse reactions				
Anaemia	532.96	479.66–586.26	Gramma	^14^
Fatigue	81.57	73.41–89.73	Gramma	^14^
Neutropenia	412	370.8–453.2	Gamma	^15^
Back pain	11.11	9.99–12.22	Gamma	^14^
Nausea	64.00	57.6–70.4	–	^14^

**Table 4:** Cost in America.

Parameter name	Base ($)	Range ($)	Distribution	Source
Medication costs				
PFS				
Talazoparib(1 mg)	163.85	131.08–196.62	–	www.yaozh.com
Talazoparib(cycle)	3440.97	2752.78–4129.16	Triangular	www.yaozh.com
Eribulin(1 mg)	547.12	545.49–654.59	Triangular	www.yaozh.com
Eribulin(cycle)	2481.76	2413.04–2,895.64	–	www.yaozh.com
Gemcitabine(200 mg)	45.98	36.784–55.176	Gramma	^16^
Gemcitabine(cycle)	1195.48	956.384–1434.576	Gramma	^16^
Capecitabine(500 mg)	3.19	2.552–3.828	Gramma	^16^
Capecitabine(cycle)	6.38	5.104–7.656	Gramma	^16^
Vinorelbine(10 mg)	11.6	9.28–13.92	–	^17^
Vinorelbine(cycle)	103.82	83.056–124.584	–	^17^
BRCA 1/2 mutation profiling	1380.17	1104.136–1656.204	Triangular	yycg.hnsggzy.com
PD				
Docetaxel (per cycle)	2228.95	375.56–2418.85	Gramma	www.yaozh.com
Prednisone (per cycle)	23.4	18.72–28.08	Gramma	www.yaozh.com
Non-pharmaceutical cost			–	
Laboratory testing (cycle)	12.67	529.20–573.30	Gramma	^14^
CT (per cycle)	828	598.00–1083.00	Gramma	^14^
Bone imaging (per cycle)	253.46	202.76–304.15	Gramma	^14^
Nursing fee (per cycle)	1617	1316–1917	Gramma	^14^
Routine follow-up (per cycle)	422	348.10–495.80	Gramma	^14^
Cost of treatment of adverse reactions				
Anaemia	1134.10	1020.59–1247.62	Gramma	^14^
Fatigue	9857.88	9168.30–10,547.47	Gramma	^14^
Neutropenia	7818	7036.2–8599.8		^18^
Back pain	12,534.53	11,280.55–13,787.46	Gamma	^14^
Nausea	719.54	465.65–1027.80	–	^14^

### Sensitivity analyses

This article uses health utility values to represent the overall level of individual health of patients. Health utility value is one of the indicators for evaluating the degree of health status, which reflects the weight between the patient's health status and complete health. The range of health utility values is between 0 and 1, with a larger number indicating better health, where 0 represents death and 1 represents complete health^19^.

This study aims to clarify the stability of model parameters through individual deterministic sensitivity analysis and probabilistic sensitivity analysis. The results of one-way deterministic sensitivity analysis are presented in the form of a tornado chart. The various bar shapes displayed in the tornado diagram represent the model output results obtained through parameters within a certain range. The longer the cylindrical strip, the greater the impact on the results. In addition, to better analyze the situation of ICER. We have determined the appropriate parameter distribution based on different types of data. Among them, the uncertainty of probability, ratio, and utility is determined by β Distribution, while the uncertainty of cost adopts γ Distribution. Monte Carlo simulation randomly extracted data from the model and simulated 1000 replicates to obtain a hypothetical queue and draw a cost–benefit curve. Corresponding WTPs were set to obtain the cost–benefit ratios of the talazoparib group and the standard treatment group, respectively. However, there is a possibility of negative costs and QALY obtained, making it difficult to analyze the average and median ICER. This study also conducted a one-way deterministic sensitivity analysis on all uncertain parameters, including transfer probability and discount rate, and controlled their variation range between ± 10% or 95% of their respective baseline values to clarify the relationship between ICER and each parameter.

Finally, this article uses NMB analysis to better analyze the situation of negative ICER. Draw cost–benefit acceptance curves using the results of NMB analysis, and set WTP thresholds of $3185/month and $19,100/month for China and the US, respectively.

### Ethics statement

All the data included in this analysis were derived from published literature and public data. No patient-identifiable data were applied or used. Therefore, institutional review board approval was not required.

## Results

### Base-case analysis

**Figure 2:**
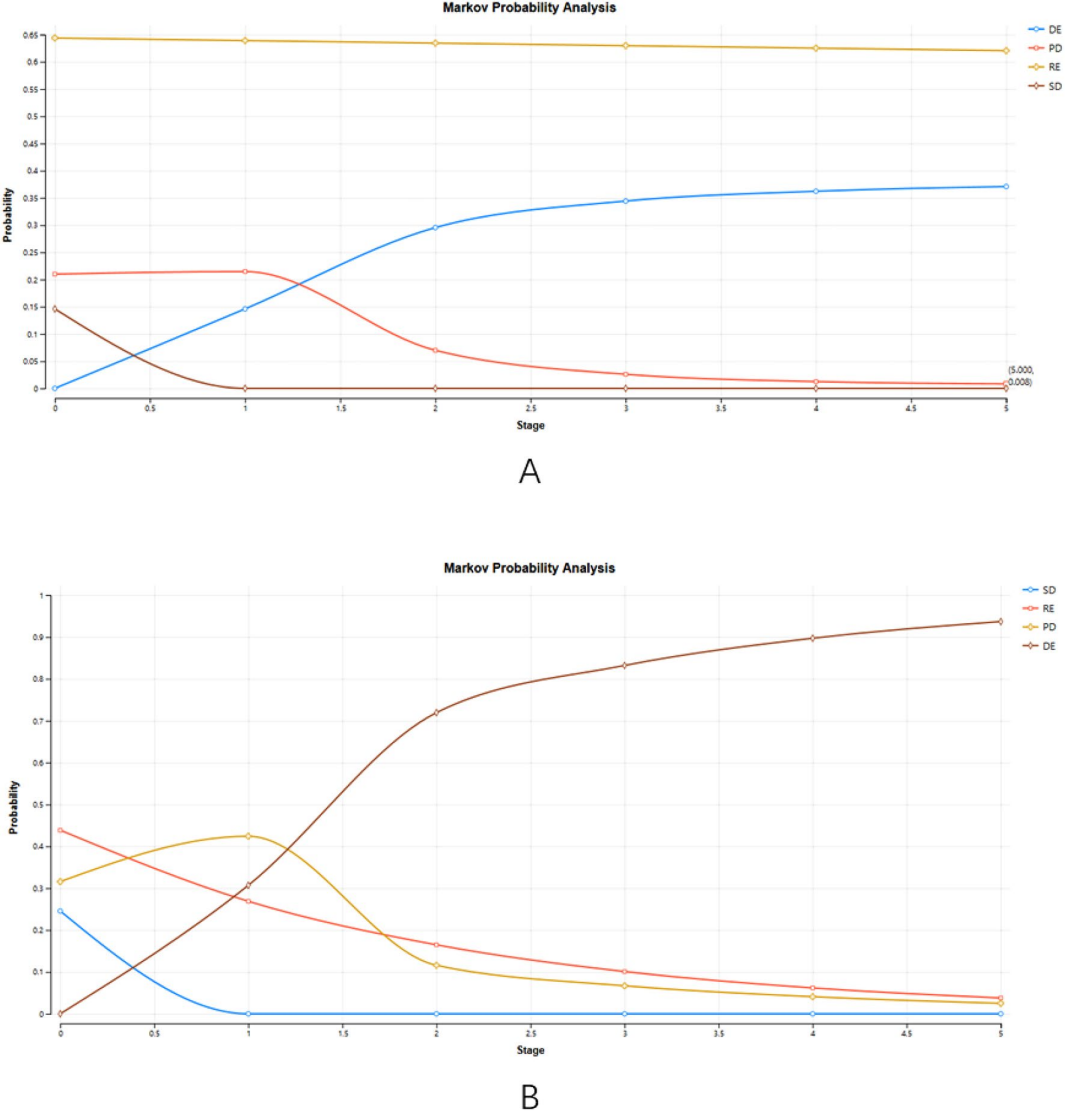
Markov Probability Analysis.

This article calculates the cumulative life cycle cost, QALY, incremental QALY, incremental cost, ICER, mortality rate, and NMB of the Talazoparib and Standard Therapy groups, respectively. The proportion of SD (0%), RE (62.1%), PD (8%), and DE (37.1%) of breast cancer patients in the talazoparib group was calculated by the Markov model (Fig. 2A). In addition, the proportions of the four distributions of Standard Therapy are SD (0%), RE (3.8%), PD (2.5%), and DE (93.7%), respectively (Fig. 2B).

Obviously, under the assumed baseline, the model results obtained show that Talazoparib is the best strategy for the advanced treatment of breast cancer. The Talazoparib group has more QALY compared to the Standard Therapy group, although it requires higher treatment costs. Due to the current dependence of China's Talazoparib on imports, the prices of Talazoparib are all uniformly based on the local Talazoparib prices in the US. The average price of Talazoparib in China and the US is 2318.65 US dollars higher than Standard Therapy. From the medical and health costs of China and the US, Talazoparib's treatment costs for advanced breast cancer patients in China and the US were $12,513.40 and $30,987.66, respectively, with 3.06 QALYs, while the standard treatment group was $8786.68 and $41,211.09, with 1.56 QALYs. The ICER values for China and the US are 2484.48 $/QALY and 6815.62 $/QALY, respectively, which are lower than the WTP values for their respective countries. The Talazoparib group achieved higher benefits in the US with lower treatment costs than the standard treatment group, while in China, although the treatment costs were higher than the standard treatment group, better benefits were achieved. So, Talazoparib has good cost-effectiveness both in China and the US. The relevant data are shown in Tables 5 and 6.

**Table 5:** The results of cost-effectiveness analysis (America).

Regimen	Cost ($)	Utility (QALY gain)	Incremental cost ($)	The incremental utility (QALY gain)	Incremental cost-utility ratio ($/QALY gain)
Talazoparib	30,987.66	3.06	–	–	–
Standard-Therapy	41,211.09	1.56	10,223.43	− 1.5	− 6815.62

**Table 6:** The results of cost-effectiveness analysis (China).

Regimen	Cost ($)	Utility (QALY gain)	Incremental cost ($)	The incremental utility (QALY gain)	Incremental cost-utility ratio ($/QALY gain)
Talazoparib	12,513.40	3.06	3726.72	1.5	2484.48
Standard-Therapy	8786.68	1.56	–	–	–

### General safety

The most common systemic adverse effects (AEs) in both groups were anemia, neutropenia, and fatigue, with grades 3–4 AEs being anemia and neutropenia. 185 patients (46%) in the talazoparib group recorded grade 3–4 anemia, while 17 patients (4%) in the standard treatment group recorded grade 3–4 anemia, which is the most common and severe AE in both groups. Neutropenia is also one of the most common AEs, with 142 patients (36%) in the talazoparib group and 28 patients (7%) in the standard treatment group. The incidence of other higher-incidence AEs, including thrombocytopenia, back pain, and fatigue^20^, is shown in Table 7.

**Table 7:** AE.

adverse events	Talazoparib		Placebo plus enzalutamide	
	All grades	Grade ≥ 3	All grades	Grade ≥ 3
Anaemia	262 (66%)	185 (46%)	70 (17%)	17 (4%)
Neutropenia	142 (36%)	73 (18%)	28 (7%)	6 (1%)
Fatigue	134 (34%)	16 (4%)	118 (29%)	8 (2%)
Back pain	88 (22%)	10 (3%)	72 (18%)	4 (1%)
Nausea	82 (21%)	2 (< 1%)	50 (12%)	3 (< 1%)

### Sensitivity analysis

**Figure 3:**
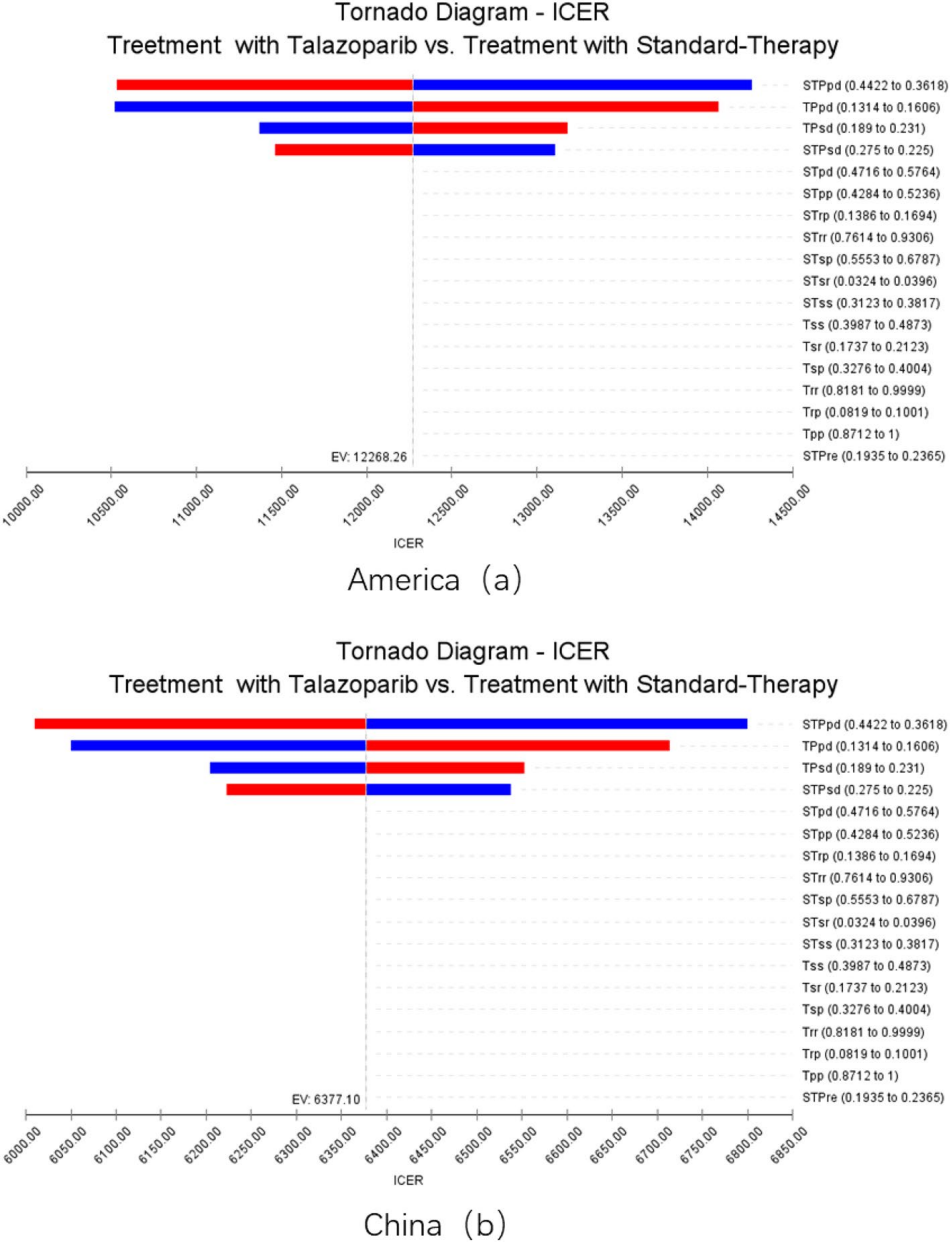
Tornado Diagram.

Through the tornado map drawn by TreeAge Pro Suite 2022, it can be seen that different parameters have different effects on the model results. The four parameters that have the greatest impact on the obtained results from tornado maps in China and the US are STPpd (probability of ST group progression to death), TPpd (probability of Talazoparib group progression to death), TPsd (probability of Talazoparib group stabilization to death), and STPsd (probability of ST group stabilization to death). Other parameters have a relatively small impact on the results. Due to sensitivity analysis, Chinese ICER is 2484.48 $/QALY, with a lower limit lower than WTP. Changes in various parameters within their sensitivity analysis range have little impact on the results of ICER. The treatment cost for the Talazoparib group in the US was $30,987.66 with a utility of 3.06 QALYs, while the standard treatment group had a cost of $41,211.09 with a utility of 1.56 QALYs. Talazoparib group achieved higher curative effect with lower cost, that is, Talazoparib is more economical in treating advanced breast cancer patients with BRCA1/2 mutation. See Fig. 3.

**Figure 4:**
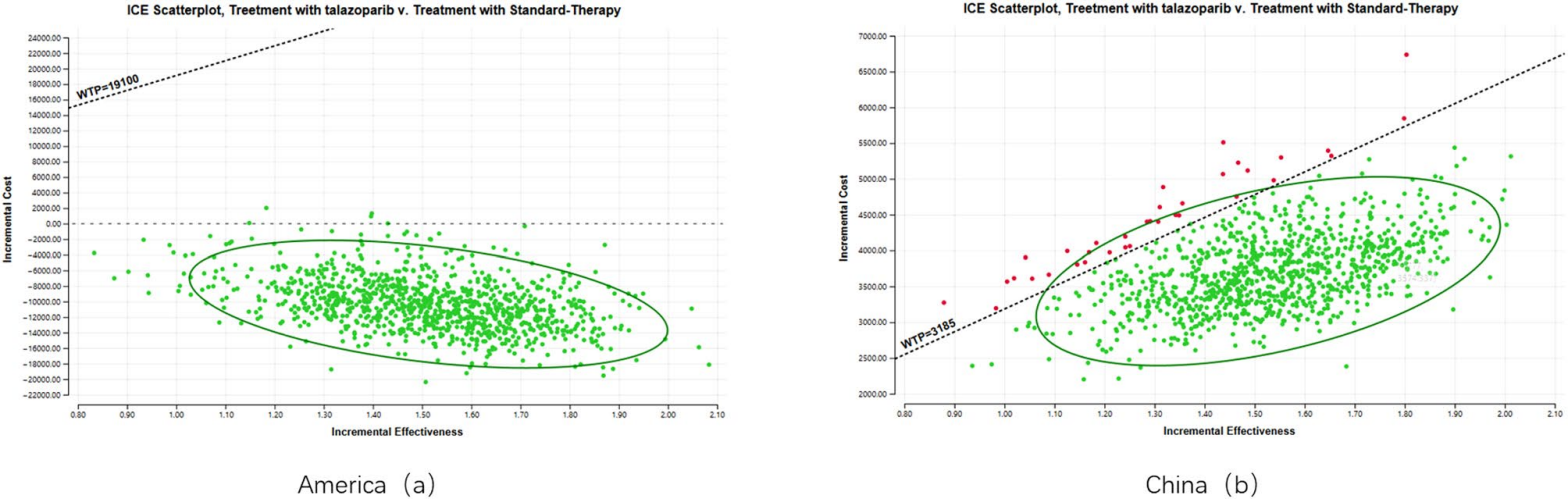
The cost-effectiveness scatter plot of the Markov model in America (**a**) and China (**b**).

In probability sensitivity analysis, this article randomly conducted 1000 Monte Carlo simulations on each parameter within the determined range of change and obtained scatter plots of Talazoparib in China and the US. The horizontal axis represents the incremental utility value, the vertical axis represents the incremental cost, and the diagonal line represents the threshold of WTP. The scattered points in the US are all below the WTP threshold line, far below the national willingness to pay value. The vast majority of scattered points in China are below the WTP threshold, below the national willingness to pay value. Therefore, Talazoparib has economic significance in the first-line treatment of patients with advanced breast cancer in China and the US. See Fig. 4.

**Figure 5:**
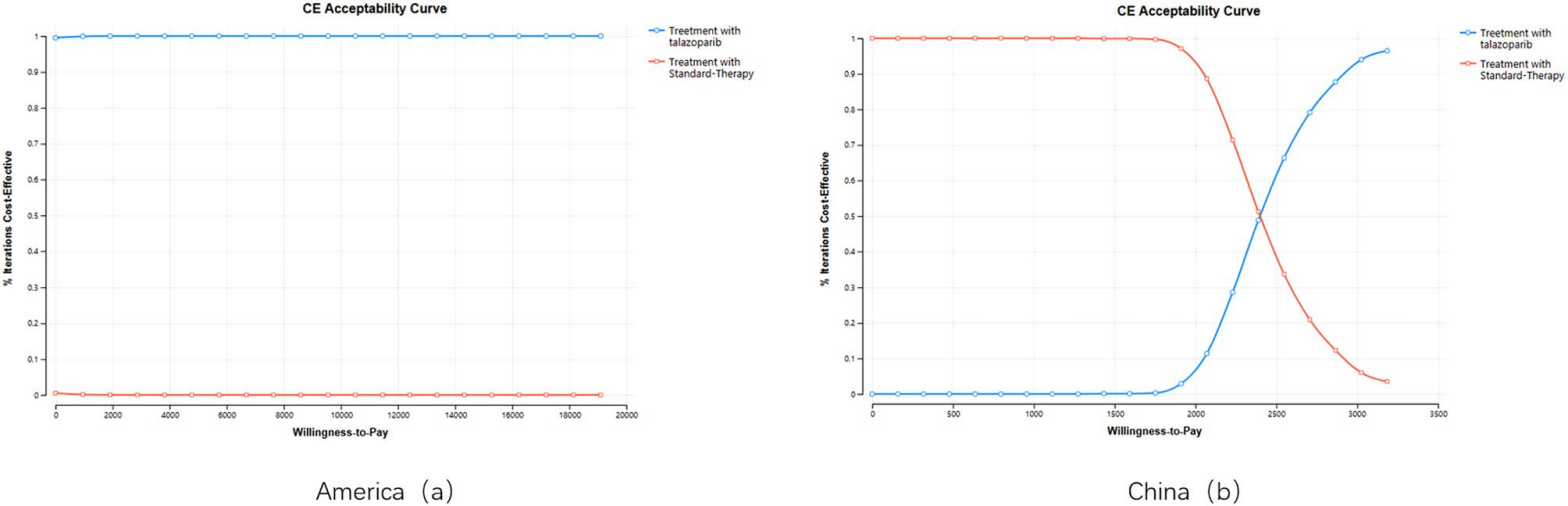
The acceptability curve of the Markov model in America (**a**) and China (**b**).

In the NMB analysis, the cost–benefit acceptability curve of patients with advanced breast cancer obtained by simulating 1000 times was drawn. As the value of WTP continues to increase, the cost-effectiveness probability of the Talazoparib group in China gradually increases after $1751, and the cost efficiency reaches 96% when the cost reaches WTP. The cost-effectiveness probability of the Talazoparib group in the US increased by 100% when the cost reached WTP. Obviously, within the WTP of China and the US, the acceptance probability of the Talazoparib group is much higher than that of the standard treatment group, making it the preferred treatment strategy. See Fig. 5.

## Discussions

BRCA1/2 gene mutation is one of the most common causes of hereditary breast cancer, which occurs in about 20% of cases with family history, and BRAC1 breast cancer is more likely to form poorly differentiated cancer^21^. The abnormal mutation of breast cancer susceptibility gene 1 or 2 in breast cancer cells can be inhibited by PARP inhibitors to repair DNA damage, thus inducing apoptosis of breast cancer cells. Talazoparib, as one of the most effective PARP inhibitors, can exert anticancer effects by effectively inhibiting the DNA single-stranded repair pathway regulated by PARP and capturing PARP in damaged DNA. According to the latest research data, this paper analyzes the cost-effectiveness of Talazoparib and the standard treatment group as BRCA1/2 mutant advanced breast cancer treatment schemes for the first time. According to the analysis of research data, compared to the standard treatment group, using Talazoparib for treatment can effectively prolong the patient's PFS, reflecting better results. The results of pharmacoeconomic research show that the talazoparib group produced a utility of 1.5 QALYs compared to the standard treatment group. Compared with the standard treatment group, the treatment cost of talazoparib group is 30,987.66$, and the ICER is 6815.62$/QALY. For Chinese patients, the treatment cost of talazoparib is 12,513.40$, and the ICER is 2484.48$/QALY. From the perspective of health services in China and the United States, our research results show that talazoparib is cost-effective in treating advanced breast cancer patients with BRCA1/2 mutations in China and the United States. In the future, patients with advanced breast cancer with BRCA1/2 mutation in China and the United States will be more inclined to choose talazoparib as the first choice of treatment, rather than the traditional Eribulin, capecitabine, gemcitabine, and vinorelbine treatment.

Through reviewing relevant literature, we found that there was a lack of economic research on the use of Talazoparib in the treatment of BRCA1/2 mutant advanced breast cancer patients. The analysis of this article is based on the medical and health systems of China and the US and includes the cost considerations of drug treatment, hospital care, auxiliary examinations, and treatment of adverse reactions. Through the analysis of the economic development situation of China and the US, the economic capacity and willingness to pay off the people of both countries are understood, and the relevant information compiled is relatively comprehensive.

However, this study still has many limitations: First, the advanced breast cancer patients in this study come from many countries and regions, and they are the data obtained in their clinical trials, not the clinical trials specifically for advanced breast cancer patients in China and the US. The study was conducted in 230 locations across 16 countries. The target group includes patients from regions other than China and the United States, which may have an impact on the research results. Although the number of patients with advanced breast cancer in this phase III clinical trial is large and the trial design is good, the basis of the model we have established all comes from this trial and depends on the effectiveness and universality of this trial. Therefore, any small deviation in this experiment may lead to bias in our research results. Secondly, the uneven economic development in different regions of China has led to significant differences in regional GDP and WTP. It is inappropriate to simply use China's per capita GDP and WTP to measure the economic effectiveness of Talazoparib for patients with advanced breast cancer in the whole country. Due to the uneven distribution of economic development and medical resources in each province, the expenses borne by the people in each province are not the same. The Markov model in the US also faces the same problem, and relevant measures should be adjusted according to local conditions. Third, the control therapy used in our study was that four antineoplastic drugs, namely, eribulin, vinorelbine, capecitabine, and gemcitabine, were randomly applied to different patients with advanced breast cancer, and the proportion of each antineoplastic drug in the standard treatment group was different. This will result in a mismatch between the costs incurred by the standard treatment group and the efficacy of the treatment, thereby exaggerating or reducing the economic effectiveness of the Talazoparib group. Fourth, the indirect costs brought by the death of patients with advanced breast cancer, such as premature death, family fragmentation, and other factors, have not been taken into account. The most important factor in sensitivity analysis is the cost of Talazoparib. To reduce the impact of Talazoparib's cost on pharmacoeconomic analysis, we expanded or reduced the scope by 10% through one-way deterministic sensitivity analysis. Finally, this study simplifies the development process of advanced breast cancer. Accurately defining the current disease status of patients in clinical practice is quite challenging. For example, in the treatment of advanced breast cancer, the adverse drug reactions and the uncertainty of tumor development and metastasis will lead to different clinical manifestations in patients.

This study is based on the medical and health systems of China and the US, which will provide a more accurate reference for doctors in China and the US to use Talazoparib in the treatment of BRCA1/2 type advanced breast cancer patients in the future, and Chinese medical insurance will provide drug economic support for negotiations on Talazoparib treatment of advanced breast cancer.

## Conclusion

From the perspective of medical and health systems in China and the US, Talazoparib has high cost-effectiveness in the treatment of advanced breast cancer patients with BRCA1/2 mutations. Therefore, Talazoparib has the opportunity to become an efficient treatment option in addition to eribulin, capecitabine, gemcitabine, and vinorelbine.

## Data Availability

The original contributions presented in the study are included in the article/Supplementary material, further inquiries can be directed to the corresponding authors.
